# Association between adaptive capacity and readiness for hospital discharge among patients with acute myocardial infarction: a cross-sectional study

**DOI:** 10.3389/fpubh.2025.1711366

**Published:** 2025-12-11

**Authors:** Wenfang Mu, Li Xu, Antai Song, Huiqin Xi, Xiyi Wang

**Affiliations:** 1 Department of Nursing, Renji Hospital, Shanghai Jiao Tong University School of Medicine, Shanghai, China; 2 Coronary Care Unit, Renji Hospital, Shanghai Jiao Tong University School of Medicine, Shanghai, China; 3 School of Nursing, Shanghai Jiao Tong University, Shanghai, China

**Keywords:** myocardial infarction, patient discharge, adaptation, capacity, association

## Abstract

**Background:**

Readiness for hospital discharge is a crucial concept in acute myocardial infarction (AMI). Positive coping and adaptation strategies are recognized as health assets that can enhance outcomes in cardiovascular disease. This study aims to investigate the relationship between readiness for hospital discharge and the patients’ adaptive capacity.

**Materials and methods:**

A cross-sectional observational study was conducted from July 2021 to March 2024 at two tertiary hospitals in Shanghai, China. A total of 373 patients diagnosed with AMI participated in this study. Data collection was conducted using a demographic information checklist and questionnaires including self-rating scale of systematic family dynamics, Chinese coping and adaptation processing–short form, and the readiness for hospital discharge scale. Statistical analyses were employed the Pearson Chi-squared test and Logistic regression.

**Results:**

The results indicate that 51.7% of AMI patients demonstrate a low level of readiness for hospital discharge. Furthermore, there is a significant positive correlation between adaptive capacity and readiness for hospital discharge (odds ratio [OR] = 1.53, 95% confidence interval [CI]: [1.01, 2.32], *p* < 0.05). Among the various dimensions of adaptive capacity, the dimensions of resourceful and focused (OR = 1.55, 95% CI [1.01, 2.36], *p* < 0.05), as well as self-initiated and knowing-based (OR = 2.26, 95% CI [1.47, 3.48], *p* < 0.001), exhibit a significant positive correlation with hospital discharge readiness, with the latter showing a stronger association.

**Conclusion:**

This study highlights that over half of patients with AMI exhibit insufficient readiness for hospital discharge. Furthermore, the patients’ adaptive capacity significantly influences their readiness for hospital discharge.

## 1 Introduction

Acute myocardial infarction (AMI) is one of the leading causes of death worldwide, accounting for approximately 9 million fatalities annually (1). The health impacts of AMI extend far beyond the acute event itself, as it continues to affect patients’ physical health, mental well-being, and overall quality of life following discharge (2). After discharge, AMI patients frequently encounter significant challenges in adjusting to family life, often exhibiting anxiety, disease-related uncertainty, and fear of recurrence. Concurrently, factors such as anxiety and fear impede the establishment of a healthy lifestyle among these patients (3–5). Meanwhile, contemporary healthcare models emphasizing shorter hospital stays and early discharge—driven by economic constraints and “enhanced recovery” pathways—have introduced early transitional care programs. However, these approaches can inadvertently leave patients insufficiently prepared to manage self-care at home, leading to poor adaptation and increased vulnerability during the transition period (6). Alarmingly, the one-year readmission rate among AMI patients can be as high as 57.6%, underscoring substantial unmet needs in post-discharge support (7).

Readiness for hospital discharge has emerged as a key determinant of successful recovery and continuity of care in AMI management (8). Evidence shows that higher levels of discharge readiness are associated with fewer unplanned readmissions, reduced cardiovascular-related emergency visits, and lower post-infarction mortality (9). Defined as a comprehensive process initiated at admission, discharge readiness involves the early identification of post-discharge care needs through systematic assessment, followed by tailored intervention planning and implementation. This continuum of care ensures that patients can transition smoothly to home or alternative care settings upon the completion of treatment (10). Importantly, readiness for discharge reflects not only the adequacy of clinical preparation and education but also patients’ capacity to adapt to changes in health status, lifestyle, and self-care responsibilities.

A growing body of research has identified numerous factors influencing discharge readiness, including the quality of discharge education, family and social support, and demographic or clinical characteristics such as age, disease severity, and length of hospitalization (11–13). Although these external and structural factors have been extensively examined, comparatively little attention has been paid to patients’ intrinsic adaptive capacity—their internal ability to self-regulate, problem-solve, and psychologically adjust to illness-related challenges (14–16). This adaptive capacity is essential for translating discharge guidance into sustained self-management and long-term recovery but remains an underexplored dimension in the literature. The limited attention to internal adaptive processes constrains the development of comprehensive models and interventions that could more effectively enhance patients’ transition from hospital to home.

Recent literature increasingly acknowledges the central role of psychological and behavioral adaptation in shaping recovery trajectories among patients with cardiovascular diseases (17, 18). Active and approach-oriented coping strategies, characterized by proactive engagement with stressors, have been shown to predict better emotional adjustment and even reduced cardiovascular mortality (12). However, the construct of adaptability has often been treated as a unitary or abstract concept in previous research, without detailed examination of its measurable dimensions or mechanisms through which it affects discharge readiness (8). Moreover, the interplay between adaptability and contextual factors—such as illness perception, subjective stress appraisal, and social environment—remains poorly understood.

This gap is both conceptual and empirical. Conceptually, adaptability has not been systematically or contextually incorporated into theoretical models of discharge readiness. Empirically, there is limited evidence identifying which specific dimensions of patients’ coping capacity most strongly predict readiness for discharge when controlling for personal, clinical, and environmental factors that influence the adaptation process. Addressing this gap is essential for advancing the theoretical understanding of discharge readiness and for developing interventions that cultivate patients’ internal strengths to enhance recovery and self-management after hospitalization. Therefore, guided by the middle-range theory of adaptation to chronic illness (19), the present study seeks to elucidate the relationship between adaptability and discharge readiness among patients with AMI. This theory conceptualizes adaptation as a dynamic, integrative process through which individuals respond to disease-related stimuli, such as treatment demands and life changes, by employing coping mechanisms that restore equilibrium and promote functional well-being. Drawing upon this framework, the current study aims to (1) identify the key dimensions of adaptability that significantly influence discharge readiness, and (2) clarify how these adaptive resources interact with personal and contextual factors to shape transitional outcomes. Ultimately, this research will generate both theoretical insights and empirical evidence to inform the design of patient-centered transitional care strategies that strengthen intrinsic adaptive capacity and optimize recovery trajectories in individuals following acute myocardial infarction.

## 2 Materials and methods

### 2.1 Study design and participants

This hospital-based, cross-sectional study was conducted between July 2021 and March 2024 in the coronary care unit of two tertiary hospitals located in the Pudong New Area and Hongkou District of Shanghai, China. The study population was patients diagnosed with AMI who were admitted to the coronary care unit of these two hospitals. Recruitment was carried out using a clustering sampling method within 24 h prior to hospital discharge to ensure that participants could accurately reflect their discharge readiness status. The inclusion criteria for patients were: having a diagnosis of first-time AMI (20), having received percutaneous coronary intervention treatment, being aged 18 years or older, and having willingness to participate. The exclusion criteria were as follows: transition to other hospitals, and disabilities that impair the ability to read the surveys or communicate with others.

The sample size was estimated using G*Power (version 3.1). Based on an expected medium effect size (OR = 1.8), a significance level (α) of 0.05, and a statistical power of 0.80, the calculated minimum sample size was 344. Initially, 430 participants were recruited to account for an anticipated dropout rate of 20%. After excluding 19 patients who were transferred to other hospitals, 10 who declined participation, 1 with severe hearing impairment, and 27 with incomplete questionnaires, the final analytical sample consisted of 373 patients diagnosed with AMI.

### 2.2 Theoretical framework

**Figure 1:**
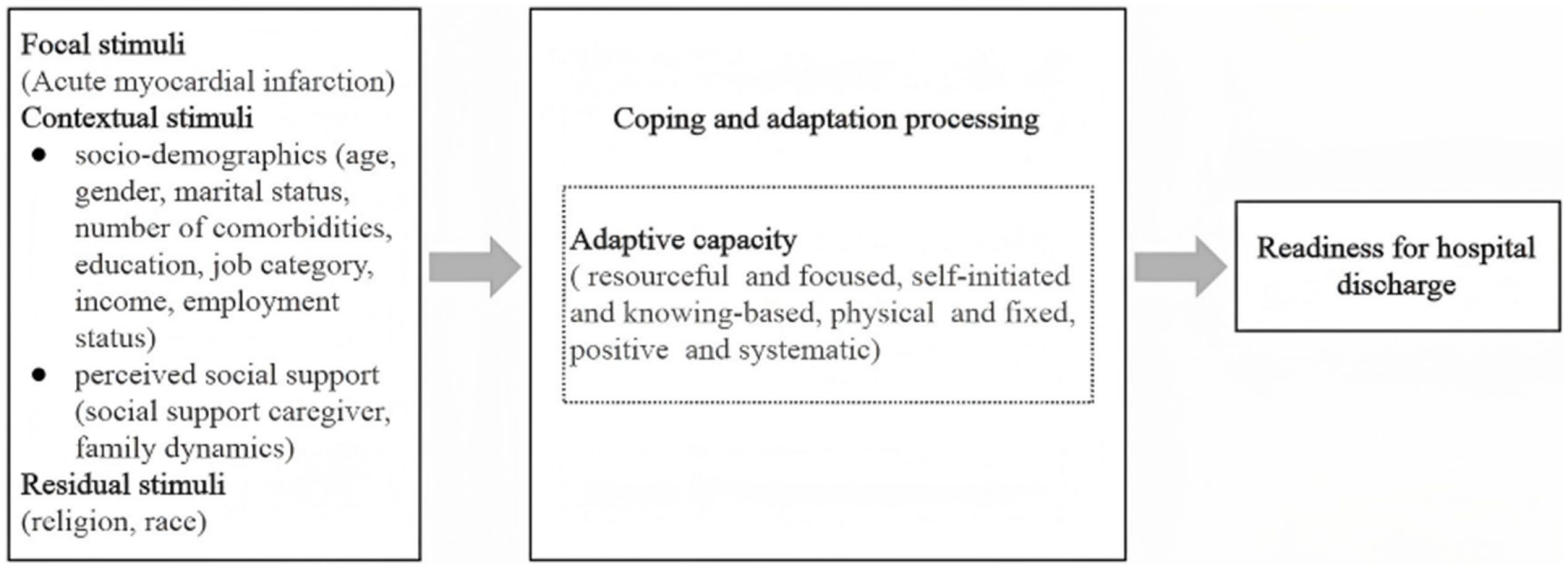
The proposed conceptual model of adaptive capacity in AMI patients based on the middle-range theory of adaptation to chronic illness.

This study is grounded in the middle-range theory of adaptation to chronic illness (19) to identify influencing factors. Within this framework, the diagnosis of AMI is conceptualized as a focal stimulus—a primary stressor that triggers adaptive processes in patients. Contextual stimuli, including demographic and contextual characteristics such as age, education level, and household income, represent the underlying personal and environmental factors that influence patients’ adaptation to illness and discharge preparedness. Together, these stimuli constitute the input component of the adaptive system. The coping and adaptation processes function as mediating mechanisms through which patients respond to these stimuli, ultimately shaping their capacity for self-regulation, emotional adjustment, and health behavior change. Discharge readiness is positioned as the adaptive outcome or output behavior that reflects the individual’s overall adjustment to illness and recovery demands. Guided by this theoretical framework, the study established a conceptual model hypothesizing the influence pathways among stimuli, coping processes, and adaptive outcomes, as illustrated in Figure 1.

### 2.3 Data collection instruments

The basic information collected included age, gender, marital status, number of comorbidities, education level, job category, household income, employment status, religion, race, and social support caregiver. The study questionnaires included family dynamics, adaptive capacity, and readiness for hospital discharge.

Family dynamics is assessed using the Self-Rating Scale of Systematic Family Dynamics (SSFD) (21). This scale was developed by Chinese scholars Yang et al. based on the theoretical frameworks and methods of systemic family therapy (22, 23). It is designed to assess individuals’ perceptions of family dynamics. The questionnaire consists of four dimensions: Family Atmosphere, Disease Concepts, System Logic, and Individualization, comprising a total of 29 items. It employs a 5-point Likert scale, with total scores ranging from 29 to 145. The standardized total score is subsequently adjusted to a scale of 0 to 100, categorizing scores into low (score ≤ 70.65) and high (score > 70.65) (8). Higher scores indicate more favorable family dynamics. The SSFD has demonstrated strong reliability (Cronbach’s α = 0.811) and cultural validity, and has been widely applied in psychological and nursing research (24, 25).

Patients’ adaptive capacity was measured using the Chinese Coping and Adaptation Processing-Short Form (C-CAPS-SF) (26). The scale is derived from the original Coping and Adaptation Processing-Short Form (CAPS-SF), initially developed by Roy et al. (27), which conceptualizes coping as a dynamic and multidimensional response to environmental and internal stimuli. This scale consists of four dimensions: resourceful and focused, self-initiated and knowing-based, physical and fixed, positive and systematic, encompassing a total of 15 items, of which 3 items necessitate reverse scoring. Items are rated on a four-point Likert scale (1 = never to 4 = always), yielding total scores from 15 to 60. Scores are standardized to a 0–100 scale, with ≤68.89 categorized as low and >68.89 as high adaptability (8). The scale demonstrates good reliability, with a McDonald’s *ω* of 0.82 for the overall scale and subscale ω values ranging from 0.56 to 0.88, indicating acceptable to strong internal consistency (26).

The Readiness for Hospital Discharge Scale is utilized to evaluate patients’ self-care abilities at the time of discharge. The scale developed by Weiss and Piacentine (28) measures readiness for hospital discharge as an adaptation outcome. The scale comprises 23 items categorized into four dimensions: physical conditions, knowledge, coping ability, and expected support, and a true/false item (excluded from scoring). Items are rated on a 10-point Likert scale, yielding total scores from 0 to 120. Standardized total scores are converted to a 0–100 scale, with ≤85 representing low and >85 representing high levels of discharge readiness (8). The original scale demonstrated excellent internal consistency (Cronbach’s α = 0.90; subscales: 0.69–0.87) (28), and the Chinese version reported similarly strong reliability (Cronbach’s α = 0.89) (29).

### 2.4 Procedures

Data collection was conducted by nurses who were nationally registered and were full-time employees at the hospital. To ensure methodological consistency and data quality, all nurses in the cardiology departments who volunteered for the study completed a three-hour standardized training session led by the chief nurse manager. The training covered participant recruitment, informed consent procedures, questionnaire administration, and ethical data handling. Eligible patients were approached within 24 h before discharge and were provided with a detailed explanation of the study objectives, procedures, and confidentiality assurances. Written informed consent was obtained prior to participation. Participants independently completed the questionnaires when possible. For those unable to do so due to physical or visual limitations, the trained research nurses read each item and response option verbatim and in a neutral tone, recording participants’ answers without leading or interpretive input. All completed questionnaires were reviewed immediately for completeness and accuracy. Data were anonymized using unique identification codes and entered into a secure electronic database. To maintain data integrity, double data entry was performed independently by two researchers, and discrepancies were resolved through cross-verification.

### 2.5 Data analysis

The data were analyzed using SPSS 26.0 and Stata/MP 17.0 software. Initially, all raw scores of the scales were converted into standardized scores to facilitate comparisons. Demographic characteristics were summarized using frequencies and percentages. The associations between categorical variables were assessed using the chi-square test. To investigate the relationship between adaptive capacity and readiness for hospital discharge, this study first conducted a multivariate logistic regression analysis. Additionally, univariate logistic regression analyses were performed to examine the crude associations between individual dimensions of adaptive capacity and the outcome. Finally, the adjusted association was derived by controlling for confounding factors. An odds ratio exceeding 1 indicates a positive correlation between the two variables, and a two-tailed test with a *p*-value of less than 0.05 is considered statistically significant.

### 2.6 Addressing potential sources of bias

Several measures were implemented to enhance internal validity and reduce bias. The cluster sampling method minimized selection bias, while the use of validated instruments and trained data collectors reduced the risk of information bias. Potential confounding was addressed by controlling for key variables in the multivariate models, thereby isolating the independent association between adaptive capacity and discharge readiness.

## 3 Results

### 3.1 Demographic characteristics

Among participants, 48.3% reported a high level of hospital discharge readiness, whereas 51.7% reported a low level. Patients with lower adaptive capacity were slightly more prevalent (52.0%) compared with those with higher adaptive capacity (48.0%). Table 1 summarizes the detailed demographic and clinical characteristics. The differences in intergroup distributions regarding age, employment status, and adaptive capacity were statistically significant (*p* < 0.05). Although education and gender were not statistically significant as independent variables, their frequent co-occurrence in sufficient configurations associated with general health outcomes supports their inclusion (30). Consequently, age, employment status, adaptive capacity, educational background, and gender were retained as the primary sociodemographic variables in the final analysis.

**Table 1:** Participants’ demographic profiles (*n* = 373).

Variables	Dimensions of variables	*n*	%	*P*
Age	≤60	118	31.6	0.004*
	>60	255	68.4	
Gender	Male	263	70.5	0.806
	Female	110	29.5	
Marital status	Single	13	3.5	0.255
	Married	339	90.9	
	Divorced	21	5.6	
Number of comorbidities	0	68	18.2	0.592
	1	186	49.9	
	≥2	119	31.9	
Education	Junior high school and below	143	38.3	0.106
	High school	131	35.1	
	College and above	99	26.5	
Job category	Intellectual labor	135	36.2	0.680
	Manual labor	163	43.7	
	None	75	20.1	
Income	≤5,000	37	9.9	0.068
	5,000–10,000	272	72.9	
	>10,000	64	17.2	
Employment status	Employed	82	22.0	0.011*
	Retired	242	64.9	
	Unemployed	49	13.1	
Social support caregiver	No	180	48.3	0.886
	Yes	193	51.7	
Family dynamic	Low	219	58.7	0.106
	High	154	41.3	
Religions	No	354	94.9	0.695
	Yes	19	5.1	
Race	Han	370	99.2	0.093
	Else	3	0.8	
Adaptive capacity	Low	194	52.0	<0.001*
	High	179	48.0	

*p < 0.05.

### 3.2 Multifactorial logistic regression analysis of adaptive capacity and readiness for hospital discharge

To investigate the relationship between adaptive capacity and readiness for hospital discharge, this study first performed a multivariate logistic regression analysis. As shown in Table 2, there is a significant positive correlation between adaptive capacity and readiness for hospital discharge (OR = 1.53, 95% CI [1.01, 2.32], *p* < 0.05), indicating that patients with higher adaptive capacity are more likely to exhibit a greater level of discharge readiness. This association remained stable even after progressively adding covariates such as educational level, age, gender, and employment status. Patients aged 60 years or younger exhibited a higher risk of discharge readiness; however, this risk lost statistical significance after incorporating employment factors. Furthermore, gender, education level, and employment status did not achieve statistical significance (*p* > 0.05).

**Table 2:** Multifactorial logistic regression analysis of adaptive capacity and readiness for hospital discharge.

Variables	Hospital discharge readiness	Hospital discharge readiness	Hospital discharge readiness	Hospital discharge readiness	Hospital discharge readiness
Adaptive capacity	1.53* [1.01, 2.32]	1.51 [1.00, 2.30]	1.53* [1.00, 2.33]	1.53* [1.01, 2.34]	1.54* [1.01, 2.35]
High school		1.38 [0.85, 2.22]	1.31 [0.81, 2.13]	1.33 [0.82, 2.16]	1.45 [0.88, 2.40]
College and above		1.66 [0.99, 2.80]	1.35 [0.78, 2.33]	1.40 [0.80, 2.43]	1.56 [0.88, 2.40]
Age			0.51** [0.32, 0.82]	0.50** [0.31, 0.80]	0.58 [0.28, 1.20]
Gender				0.85 [0.53, 1.36]	0.85 [0.53, 1.37]
Retired					1.20 [0.52, 2.73]
Unemployed					1.81 [0.91, 3.59]

Exponentiated coefficients; 95% confidence intervals in brackets.

*p < 0.05,**p < 0.01.

### 3.3 Univariate analysis of each dimension of adaptive capacity and readiness for hospital discharge

Subsequently, a univariate regression analysis was conducted on the dimensions of adaptability, with the results presented in Table 3. The dimensions of resourceful and focuse (OR = 1.64, 95% CI [1.08, 2.49], *p* < 0.05), as well as self-initiated and knowing-based (OR = 2.03, 95% CI [1.34, 3.08], *p* < 0.001), exhibit a significant positive correlation with readiness for hospital discharge, with the latter showing a stronger association. Although “physical and fixed” and “positive and systematic” demonstrate a positive trend, they do not achieve statistical significance (*p* > 0.05).

**Table 3:** Univariate analysis of each dimension of adaptive capacity and hospital discharge readiness.

Variables	Hospital discharge readiness	Hospital discharge readiness	Hospital discharge readiness	Hospital discharge readiness
(1) Resourceful and focused	1.64* [1.08, 2.49]			
(2) Self-initiated and knowing-based		2.03*** [1.34, 3.08]		
(3) Physical and fixed			1.26 [0.83, 1.91]	
(4) Positive and systematic				1.31 [0.87, 1.97]

Exponentiated coefficients; 95% confidence intervals in brackets.

*p < 0.05, **p < 0.01, ***p < 0.001.

### 3.4 The adjusted model after controlling for confounding factors

After controlling for confounding factors, an adjusted model was established. The results indicated a correlation between the two dimensions of adaptability and patients’ readiness for discharge. Specifically, self-initiated and knowledge-based adaptability exhibited the strongest independent association (OR = 2.26, 95% CI [1.47, 3.48], *p* < 0.001). Although the association for resourceful and focused adaptability showed a slight weakening, it remained statistically significant (OR = 1.55, 95% CI [1.01, 2.36], *p* < 0.05). Further detailed information can be found in Table 4.

**Table 4:** The adjusted model after controlling for confounding factors.

Variables	Hospital discharge readiness	Hospital discharge readiness	Hospital discharge readiness	Hospital discharge readiness
Resourceful and focused	1.55* [1.01, 2.36]			
Self-initiated and knowing-based		2.26*** [1.47, 3.48]		
Physical and fixed			1.22 [0.80, 1.88]	
Positive and systematic				1.32 [0.86, 2.00]
High school	1.43 [0.87, 2.3]	1.40 [0.84, 2.33]	1.43 [0.87, 2.35]	1.41 [0.85, 2.32]
College and above	1.54 [0.87, 2.73]	1.59 [0.89, 2.84]	1.58 [0.89, 2.79]	1.55 [0.88, 2.75]
Age	0.60 [0.29, 1.22]	0.49 [0.24, 1.03]	0.60 [0.29, 1.23]	0.58 [0.28, 1.19]
Gender	0.88 [0.54, 1.41]	0.87 [0.53, 1.41]	0.87 [0.54, 1.40]	0.86 [0.53, 1.38]
Retired	1.18 [0.52, 2.69]	1.10 [0.48, 2.54]	1.22 [0.54, 2.77]	1.20 [0.53, 2.73]
Unemployed	1.77 [0.89, 3.52]	1.71 [0.85, 3.42]	1.83 [0.92, 3.63]	1.79 [0.90, 3.55]

Exponentiated coefficients; 95% confidence intervals in brackets.

*p < 0.05, **p < 0.01, ***p < 0.001.

## 4 Discussion

This study highlights that 51.7% demonstrated low discharge readiness, highlighting that a substantial proportion of AMI patients are inadequately prepared for hospital-to-home transitions. This prevalence is slightly higher than reported in previous studies. In comparison, a cross-sectional study involving 102 AMI patients in Wrocław, Poland, found that 47.06% exhibited low readiness for hospital discharge, 27.45% were in a moderate state, and 25.49% reported a high level of readiness for discharge (31). Similarly, another cross-sectional study conducted with a selected group of 242 patients in the Silesian Voivodeship, Poland, revealed that 40.6% of AMI patients exhibited low hospital discharge readiness, 42.9% demonstrated a moderate level, and only 16.5% reported a high level of discharge readiness (32). A likely explanation is that patients in the present study were discharged directly from the coronary care unit, bypassing general wards. Patients discharged directly from intensive care settings often have higher illness severity and shorter recovery periods, resulting in lower perceived readiness. These findings underscore an urgent need for targeted interventions to enhance discharge preparedness. Notably, this study employed a binary classification of readiness (high vs. low), validated clinically by our team. While this differs from previous three-level categorizations, the consistent pattern across studies—substantial proportions of patients with inadequate preparedness—confirms that low discharge readiness is a widespread and persistent clinical challenge.

This study found that fewer than half (48%) of AMI patients demonstrated high adaptive capacity, highlighting the substantial variability in patients’ ability to cope with acute illness and its consequences. Evidence suggests that patients who possess strong health beliefs, a sense of control, and confidence in themselves, their families, and healthcare professionals are better able to develop adaptive coping strategies. These strategies facilitate adherence to treatment regimens and support sustained participation in cardiac rehabilitation programs (3, 33). Furthermore, patients with high adaptability perceive their illness as a positive event, a turning point that provides them with the opportunity to reconsider their daily lives, alter their physical and dietary habits to better promote health, and ultimately gain benefits (34, 35).

Consistent with theoretical expectations, adaptive capacity was positively associated with readiness for hospital discharge (OR = 1.53, 95% CI [1.01, 2.32]). Prior studies have shown that adaptive capacity enables patients to develop self-care competence and confidence, reduces perceived transitional difficulties, and enhances discharge readiness (36). Conversely, patients with lower acceptance of their illness and adaptability tend to experience longer hospital stays, poorer discharge readiness, and are more likely to encounter adverse events, resulting in worse physical and mental health outcomes (31). Furthermore, patients who experience high stress and maladaptation during hospitalization face significant challenges in managing their health after discharge, leading to greater post-discharge coping difficulties (11). In contrast to maladaptive characteristics such as avoidance and loneliness, adaptable patients report lower levels of depressive symptoms and post-traumatic stress symptoms 3 and 12 months after discharge (13). Therefore, enhancing adaptive capacity and increasing recovery confidence can improve readiness for hospital discharge in AMI patients, allowing them to adjust to the hospital environment more swiftly, complete high-quality treatment and care during a brief hospitalization, and reduce post-discharge coping challenges.

Among the four dimensions of adaptive capacity, resourceful and focused coping behaviors were positively associated with discharge readiness (OR = 1.55, 95% CI [1.01, 2.36]). This dimension reflects the effective mobilization and integration of external resources through planning, problem-solving, and purposeful action, enabling patients to navigate specific challenges during hospitalization and post-discharge recovery. Evidence indicates that access to adequate resources significantly enhances patients’ ability to adapt to illness and adversity, whereas insufficient resources may exacerbate stress and compromise health outcomes (37, 38). Clinically, healthcare professionals should prioritize resource provision throughout the hospitalization process (9), including assessment of patients’ concerns and educational needs, delivery of personalized education, medication reconciliation, care coordination, and the development of individualized discharge plans encompassing treatment goals and follow-up instructions (39). Furthermore, the hospital-community collaborative transition program has proven effective in reducing readmissions and enhancing care transitions (40). With the support of digital health technologies, such as telemedicine, remote monitoring, telephone support, and mobile applications, patients can be assisted in their coping and adaptation processes. This support enhances their self-care skills and ultimately accelerates their recovery both during and after hospitalization (41–44).

Similarly, the self-initiated and knowledge-based dimension demonstrated the strongest association with discharge readiness (OR = 2.26, 95% CI [1.47, 3.48]), highlighting that patients who actively engage in managing their illness and applying health-related knowledge are more than twice as likely to achieve high readiness. It is crucial for individuals who are self-initiated and knowledge-based to actively engage as leaders in managing their illness (26). Supporting evidence from related studies corroborates this notion. To bolster self-care and improve coping and adaptation, patients must independently initiate and sustain their activities. However, the efficacy of these self-initiated activities is contingent upon the patients’ strengths and capabilities (44–46). These findings underscore the importance of personal-level interventions, including mindfulness-based programs and positive psychological strategies, which enhance internal resilience and facilitate adaptive coping. Furthermore, the use of patient profiling through cluster analysis allows for tailored interventions that account for individual personality traits and risk factors, optimizing readiness for discharge (47). Overall, these results suggest that improving hospital discharge preparedness in AMI patients requires a dual focus on internal adaptive resources, such as resilience and proactive self-management, and external supports, including accessible resources and structured transitional care, to ensure effective coping and successful recovery.

## 5 Limitations and strengths

This study has several limitations. First, it was conducted in two tertiary hospitals in Shanghai, which may limit the generalizability of findings to other regions, healthcare settings, or patient populations. Second, the cross-sectional design precludes causal inferences, and longitudinal studies are needed to examine how adaptive capacity influences discharge readiness over time. Third, while adaptive capacity was the primary focus, other psychosocial, environmental, and system-level factors that may influence discharge readiness were not assessed and warrant further investigation.

Despite these limitations, this study has notable strengths. It is theoretically grounded, guided by the middle-range theory of adaptation to chronic illness, which provides a robust framework for interpreting the relationship between adaptive capacity and discharge readiness. The study employed validated instruments, standardized data collection procedures, and rigorous statistical analyses, enhancing the reliability and internal validity of the findings. Importantly, the identification of specific adaptive capacity dimensions offers actionable insights for designing targeted, patient-centered interventions to improve discharge readiness among patients with AMI.

## 6 Conclusion

This study highlights that over half of patients with AMI exhibit insufficient readiness for hospital discharge, emphasizing a critical gap in transitional care. Adaptive capacity emerged as a key determinant of discharge readiness, with resourceful and focused, and self-initiated and knowledge-based behaviors showing the strongest influence. These findings underscore that successful hospital-to-home transitions depend not only on adequate external resources but also on strengthening patients’ intrinsic adaptive skills. Clinically, interventions that simultaneously enhance patients’ resilience, self-management abilities, and access to tailored support are essential to optimize discharge preparedness, reduce post-discharge complications, and improve overall recovery trajectories among patients with AMI.

## Data Availability

The raw data supporting the conclusions of this article will be made available by the authors, without undue reservation.

## References

[1] WHO. Health emergency and disaster risk management framework (2019). Available online at: https://iris.who.int/handle/10665/326106 (Accessed June 1, 2025).

[2] Radaideh A Rababah JA Al-Hammouri MM Ta'an W Suliman M. The association of health literacy with the quality of discharge planning and readiness for hospital discharge in Jordanian acute myocardial infarction patients. J Clin Nurs. (2025) 34:3745–52. doi: 10.1111/jocn.17543, 39520045

[3] Wang X Chen D Zou P Zhang H Qiu X Xu L . Understanding adaptive tasks in cardiac rehabilitation among patients with acute myocardial infarction: a qualitative study. Ann Med. (2024) 56:2311227. doi: 10.1080/07853890.2024.2311227, 38306095 PMC10840589

[4] Vidal-Almela S Czajkowski B Prince SA Chirico D Way KL Pipe AL . Lessons learned from community- and home-based physical activity programs: a narrative review of factors influencing women's participation in cardiac rehabilitation. Eur J Prev Cardiol. (2021) 28:761–78. doi: 10.1177/2047487320907748, 33611528

[5] Solano-Ruiz M Freitas GF Ugarte-Gurrutxaga MI Gómez-Cantarino S Siles-González J. Men's positive and negative experiences following acute myocardial infarction. Int J Environ Res Public Health. (2021) 18:1053. doi: 10.3390/ijerph18031053, 33504041 PMC7908433

[6] Fuller TE Pong DD Piniella N Pardo M Bessa N Yoon C . Interactive digital health tools to engage patients and caregivers in discharge preparation: implementation study. J Med Internet Res. (2020) 22:e15573. doi: 10.2196/15573, 32343248 PMC7218608

[7] Butler J Hammonds K Talha KM Alhamdow A Bennett MM Bomar JVA . Incident heart failure and recurrent coronary events following acute myocardial infarction. Eur Heart J. (2025) 46:1540–50. doi: 10.1093/eurheartj/ehae885, 39874177 PMC12011519

[8] Chen D Xu L Lee G Wang X. Complex determinants of hospital discharge readiness among patients with acute myocardial infarction: a fuzzy-set qualitative comparative analysis. Int J Nurs Stud. (2025) 170:105172. doi: 10.1016/j.ijnurstu.2025.105172, 40752300

[9] Homem F Reveles A Amaral A Coutinho V Gonçalves L. Improving transitional care after acute myocardial infarction: a scoping review. Health Care Sci. (2024) 3:312–28. doi: 10.1002/hcs2.116, 39479273 PMC11520247

[10] Cherlin EJ Curry LA Thompson JW Greysen SR Spatz E Krumholz HM . Features of high quality discharge planning for patients following acute myocardial infarction. J Gen Intern Med. (2013) 28:436–43. doi: 10.1007/s11606-012-2234-y, 23263917 PMC3579981

[11] Marsall M Hornung T Bäuerle A Weiss ME Teufel M Weigl M. Coping difficulties after inpatient hospital treatment: validity and reliability of the German version of the post-discharge coping difficulty scale. J Patient Rep Outcomes. (2024) 8:125. doi: 10.1186/s41687-024-00806-9, 39499458 PMC11538096

[12] Svensson T Inoue M Sawada N Yamagishi K Charvat H Saito I . Coping strategies and risk of cardiovascular disease incidence and mortality: the Japan public health center-based prospective study. Eur Heart J. (2016) 37:890–9. doi: 10.1093/eurheartj/ehv724, 26746633

[13] Zuccarella-Hackl C Jimenez-Gonzalo L von Känel R Princip M Jellestad L Langraf-Meister RE . Positive psychosocial factors and the development of symptoms of depression and posttraumatic stress symptoms following acute myocardial infarction. Front Psychol. (2023) 14:1302699. doi: 10.3389/fpsyg.2023.1302699, 38111867 PMC10725949

[14] Thoma JE Waite MA. Experiences of nurse case managers within a central discharge planning role of collaboration between physicians, patients and other healthcare professionals: a sociocultural qualitative study. J Clin Nurs. (2018) 27:1198–208. doi: 10.1111/jocn.14166, 29148105

[15] Tuna S Pakyüz S. The effectiveness of planned discharge education on health knowledge and beliefs in patients with acute myocardial infarction: a randomized controlled trial. Ir J Med Sci. (2022) 191:691–8. doi: 10.1007/s11845-021-02601-7, 33728530

[16] Kosobucka A Michalski P Pietrzykowski Ł Kasprzak M Fabiszak T Felsmann M . The impact of readiness to discharge from hospital on adherence to treatment in patients after myocardial infarction. Cardiol J. (2022) 29:582–90. doi: 10.5603/CJ.a2020.0005, 32037501 PMC9273254

[17] Zhao JY Jiang JF Shi SY Su XQ Que WQ Zhu LL . Mediating effects of resilience and perceived social support between gratitude and the adaptability to return-to-work in cancer patients. J Adv Nurs. (2025) 1–10. doi: 10.1111/jan.16966 40211591

[18] Owusu BA Ofori-Boateng P Doku DT. Coping and adaptation strategies among young persons living with type 1 diabetes and their caregivers: textual and photovoice analyses. BMC Public Health. (2023) 23:1684. doi: 10.1186/s12889-023-16573-z, 37653475 PMC10472693

[19] Wang XY Ye ZH. Development of a middle-range theory of adaptation to chronic illness based on the Roy's model. Chin J Nurs. (2021) 56:1193–200. doi: 10.3761/j.issn.0254-1769.2021.08.012

[20] Collet JP Thiele H Barbato E Barthélémy O Bauersachs J Bhatt DL . 2020 ESC guidelines for the management of acute coronary syndromes in patients presenting without persistent ST-segment elevation. Eur Heart J. (2021) 42:1289–367. doi: 10.1093/eurheartj/ehaa575, 32860058

[21] Yang JZ Kang CY Zhao XD Xu XF. The self-rating inventory of systematic family dynamics: development, reliability and validity. Chin J Clin Psyc 2002, 263–265. Available online at: http://www.clinicalpsychojournal.com/Magazine/Show.aspx?ID=141259

[22] Simon GM. A revisionist rendering of structural family therapy. J Marital Fam Ther. (1995) 21:17–26. doi: 10.1111/j.1752-0606.1995.tb00135.x

[23] Sluzki CE. Process, structure and world views: toward an integrated view of systemic models in family therapy. Fam Process. (1983) 22:469–76. doi: 10.1111/j.1545-5300.1983.00469.x, 6677518

[24] Yang Z Shang Y Liang Y Zhang H Yang Y Wang Y . The quality of life and its relationship with systemic family dynamics and mental health in senior high school students from Shaanxi, China. Front Public Health. (2022) 10:833561. doi: 10.3389/fpubh.2022.833561, 35433624 PMC9008304

[25] Shi J Tao Y Yan C Zhao X Wu X Zhang T . A study on the correlation between family dynamic factors and depression in adolescents. Front Psych. (2022) 13:1025168. doi: 10.3389/fpsyt.2022.1025168, 36762296 PMC9902595

[26] Wang X Tang L Howell D Shao J Qiu R Zhang Q . Psychometric testing of the Chinese version of the coping and adaptation processing scale-short form in adults with chronic illness. Front Psychol. (2020) 11:1642. doi: 10.3389/fpsyg.2020.01642, 32849006 PMC7396654

[27] Roy C Bakan G Li Z Nguyen TH. Coping measurement: creating short form of coping and adaptation processing scale using item response theory and patients dealing with chronic and acute health conditions. Appl Nurs Res. (2016) 32:73–9. doi: 10.1016/j.apnr.2016.06.002, 27969056

[28] Weiss ME Piacentine LB. Psychometric properties of the readiness for hospital discharge scale. J Nurs Meas. (2006) 14:163–80. doi: 10.1891/jnm-v14i3a002, 17278337

[29] Lin YH Kao CC Huang AM Chi MT Chou FH. Psychometric testing of the chinese version of the readiness for hospital discharge scale. Hu Li Za Zhi. (2014) 61:56–65. doi: 10.6224/JN.61.4.56, 25116315

[30] Hao C Guo D Ren H Wang X Qiao Y Qiu L. The relationship between social capital and health from a configuration perspective: an evidence from China. BMC Public Health. (2023) 23:1611. doi: 10.1186/s12889-023-16547-1, 37612596 PMC10463615

[31] Hydzik P Kolarczyk E Kustrzycki W Kubielas G Kałużna-Oleksy M Szczepanowski R . Readiness for discharge from hospital after myocardial infarction: a cross-sectional study. Int J Environ Res Public Health. (2021) 18:6937. doi: 10.3390/ijerph18136937, 34203516 PMC8297344

[32] Kolarczyk E Witkowska A Szymiczek M Młynarska A. The variables of the readiness for Discharge from Hospital in Patients after myocardial infarction. Int J Environ Res Public Health. (2023) 20:1582. doi: 10.3390/ijerph20021582, 36674337 PMC9867362

[33] Bohplian S Bronas UG. Motivational strategies and concepts to increase participation and adherence in cardiac rehabilitation: AN INTEGRATIVE REVIEW. J Cardiopulm Rehabil Prev. (2022) 42:75–83. doi: 10.1097/HCR.0000000000000639, 34753873

[34] Dreyer RP Pavlo AJ Hersey D Horne A Dunn R Norris CM . "is my heart healing?" a Meta-synthesis of patients' experiences after acute myocardial infarction. J Cardiovasc Nurs. (2021) 36:517–30. doi: 10.1097/JCN.0000000000000732, 32740224

[35] Fredriksson-Larsson U Alsén P Karlson BW Brink E. Fatigue two months after myocardial infarction and its relationships with other concurrent symptoms, sleep quality and coping strategies. J Clin Nurs. (2015) 24:2192–200. doi: 10.1111/jocn.12876, 25988847 PMC4744703

[36] Ronaldson A Molloy GJ Wikman A Poole L Kaski JC Steptoe A. Optimism and recovery after acute coronary syndrome: a clinical cohort study. Psychosom Med. (2015) 77:311–8. doi: 10.1097/PSY.0000000000000155, 25738438 PMC4396437

[37] Felix AS Shisler R Nolan TS Warren BJ Rhoades J Barnett KS . High-effort coping and cardiovascular disease among women: a systematic review of the John Henryism hypothesis. J Urban Health. (2019) 96:12–22. doi: 10.1007/s11524-018-00333-1, 30506136 PMC6430283

[38] Aspinwall LG Tedeschi RG. The value of positive psychology for health psychology: progress and pitfalls in examining the relation of positive phenomena to health. Ann Behav Med. (2010) 39:4–15. doi: 10.1007/s12160-009-9153-0, 20091429

[39] Kaldal A Tonstad S Jortveit J. Long-term hospital-based secondary prevention of coronary artery disease: a randomized controlled trial. BMC Cardiovasc Disord. (2021) 21:600. doi: 10.1186/s12872-021-02426-3, 34915839 PMC8679993

[40] Cao XY Tian L Chen L Jiang XL. Effects of a hospital-community partnership transitional program in patients with coronary heart disease in Chengdu, China: a randomized controlled trial. Jpn J Nurs Sci. (2017) 14:320–31. doi: 10.1111/jjns.12160, 28150384

[41] Ruschel KB Rados DR Furtado MV Batista JDL Katz N Harzheim E . Transition of care of stable ischaemic heart disease patients from tertiary to primary care with telemedicine support: randomized noninferiority clinical trial. J Telemed Telecare. (2022) 28:52–7. doi: 10.1177/1357633X20906648, 32188310

[42] Su JJ Yu DS. Effects of a nurse-led eHealth cardiac rehabilitation programme on health outcomes of patients with coronary heart disease: a randomised controlled trial. Int J Nurs Stud. (2021) 122:104040. doi: 10.1016/j.ijnurstu.2021.104040, 34333211

[43] Ivers NM Schwalm JD Bouck Z McCready T Taljaard M Grace SL . Interventions supporting long term adherence and decreasing cardiovascular events after myocardial infarction (ISLAND): pragmatic randomised controlled trial. BMJ. (2020) 369:m1731. doi: 10.1136/bmj.m1731, 32522811 PMC7284284

[44] Kaya U Dal Yılmaz Ü. Ideal suggestions for discharge training and telephone counseling of patients with coronary artery bypass graft surgery: a randomized controlled and experimental study. J Korean Med Sci. (2022) 37:e269. doi: 10.3346/jkms.2022.37.e269, 36065653 PMC9444573

[45] Kubzansky LD Huffman JC Boehm JK Hernandez R Kim ES Koga HK . Positive psychological well-being and cardiovascular disease: JACC health promotion series. J Am Coll Cardiol. (2018) 72:1382–96. doi: 10.1016/j.jacc.2018.07.042, 30213332 PMC6289282

[46] Zuccarella-Hackl C Princip M Auschra B Meister-Langraf RE Barth J von Känel R. Association of positive psychological well-being with circulating inflammatory markers: a systematic review and meta-analysis. Neurosci Biobehav Rev. (2023) 150:105186. doi: 10.1016/j.neubiorev.2023.105186, 37076058

[47] Doron J Trouillet R Maneveau A Ninot G Neveu D. Coping profiles, perceived stress and health-related behaviors: a cluster analysis approach. Health Promot Int. (2015) 30:88–100. doi: 10.1093/heapro/dau090, 25324530

